# Personal

**Published:** 1896-06

**Authors:** 


### Personal.

Drs. L. S. McMurtry, of Louisville, and Charles A. L. Reed, of Cincinnati, have been appointed honorary presidents of the International Periodic Congress of Gynecology and Obstetrics, which holds its meeting at Geneva during the first week in September, 1896. Dr. McMurtry will sail from New York on the French line July 18th.

---

Dr. Wm. Warren Potter, of Buffalo, has been appointed vice-president of the auxiliary committee of the second Pan-American Medical Congress for Buffalo and vicinity. This congress convenes in the City of Mexico in November of this year.

---

Dr. Irving M. Snow and Mrs. Julia F. Snow, of Franklin street, Buffalo, sailed, May 27, 1896, on the American liner City of New York, for a month's stay in England. They will return early in July.

---

Dr. J. W. Grosvenor, of Buffalo, was elected vice-president of the American Academy of Medicine during its recent annual meeting at Atlanta.

---

Dr. and Mrs. Alphonse Dagenais and Miss Blanche Dagenais, of Virginia street, Buffalo, will spend the summer abroad.

---

Dr. and Mrs. Lucien Howe, of Delaware avenue, Buffalo, have returned from a trip abroad.

